# Accessible Communication Tools for Surgical Site Infection Monitoring and Prevention in Joint Reconstruction: Feasibility Study

**DOI:** 10.2196/periop.7874

**Published:** 2018-01-17

**Authors:** Keyin Lu, Christopher J Chermside-Scabbo, Nikolas Evan Marino, Angela Concepcion, Craig Yugawa, Bola Aladegbami, Theodora Paar, Theresa A St John, Will Ross, John C Clohisy, John P Kirby

**Affiliations:** ^1^ School of Medicine Saint Louis University St Louis, MO United States; ^2^ School of Medicine Washington University in Saint Louis St Louis, MO United States; ^3^ Department of Orthopaedics Washington University in Saint Louis St Louis, MO United States; ^4^ Department of Surgery Washington University in Saint Louis St Louis, MO United States; ^5^ Department of Nephrology Washington University in Saint Louis St Louis, MO United States

**Keywords:** communication tool, decolonization, mobile health, surgical site infection, automated, messaging

## Abstract

**Background:**

The National Surgical Quality Improvement Program logs surgical site infections (SSIs) as the most common cause of unplanned postoperative readmission for a variety of surgical interventions. Hospitals are making significant efforts preoperatively and postoperatively to reduce SSIs and improve care. Telemedicine, defined as using remote technology to implement health care, has the potential to improve outcomes across a wide range of parameters, including reducing SSIs.

**Objective:**

The purpose of this study was to assess the feasibility and user satisfaction of two automated messaging systems, EpxDecolonization and EpxWound, to improve perioperative care in a quality improvement project for patients undergoing total joint replacement.

**Methods:**

We designed two automated text messaging and calling systems named EpxDecolonization, which reminded patients of their preoperative decolonization protocol, and EpxWound, which monitored pain, wound, and fever status postoperatively. Daily patient responses were recorded and a post-usage survey was sent out to participants to assess satisfaction with the systems.

**Results:**

Over the 40-week study period, 638 and 642 patients were enrolled in EpxDecolonization (a preoperative decolonization reminder) and EpxWound (a postoperative surgical site infection telemonitoring system), respectively. Patients could be enrolled in either or both EpxDecolonization and EpxWound, with the default option being dual enrollment. The proportion of sessions responded to was 85.2% for EpxDecolonization and 78.4% for EpxWound. Of the 1280 patients prescribed EpxWound and EpxDecolonization, 821 (64.14%) fully completed the postoperative system satisfaction survey. The median survey score (scale 1-9) was 9 for patient-rated overall care and 8 for whether the telemonitoring systems improved patient communication with providers. The majority of patients (69.0%, 566/821) indicated that the systems sent out an ideal number of messages (not too many, not too few).

**Conclusions:**

EpxDecolonization and EpxWound demonstrated high response rates and improved patient-rated communication with providers. These preliminary data suggest that these systems are well tolerated and potentially beneficial to both patients and providers. The systems have the potential to improve both patient satisfaction scores and compliance with preoperative protocols and postoperative wound monitoring. Future efforts will focus on testing the sensitivity and specificity of alerts generated by each system and on demonstrating the ability of these systems to improve clinical quality metrics with more authoritative data.

## Introduction

According to the National Surgical Quality Improvement Program of the American College of Surgeons, surgical site infections (SSIs) were the most common cause (1.1%) of unplanned surgical 30-day readmissions overall in 2012 for 346 US hospitals [1]. The cost of treating an SSI can be between US $27,000 and US $40,000 per infection per patient. In particular, SSIs for orthopedic patients result in longer hospital stays, higher readmission rates, and up to quadruple the health care costs due to prolonged antibiotics and additional hardware revisions [2,3]. It is estimated that by 2020 there will be at least 70,000 total hip and knee arthroplasty revision surgeries due to deep SSIs at a cost of US $1.62 billion annually [4]. Readmission rates are now an important quality metric for hospitals and the Centers for Medicare and Medicaid Services are focusing on identifying the causes for readmission in an effort to improve quality of care and control costs. Given the importance of SSIs in postsurgery readmissions and their clinical impact on patients receiving implanted orthopedic hardware, we wanted to study how enhanced telemedicine techniques could prevent, detect, and treat SSIs earlier and at reduced system costs.

Telemedicine, the use of technology to deliver health care remotely [5], shows promise in improving prevention and detection of SSIs. Medication adherence and patient outcomes have been shown to improve with interventions that include reminders [6]. In a survey querying patients’ experiences with postoperative self-management of wounds after surgery, patients reported concern about the efficacy of self-monitoring and whether health care providers would be accessible if wound issues developed [7]. Despite these initial concerns, the majority of patients expressed openness toward a mobile intervention. Although there are currently many digital platforms for telemedicine that include email or health portals, those both require reliable Internet access or “smart” mobile phones. A text message-based intervention seems particularly promising due to the wide and convenient availability of cell phones. Short message service (SMS) text messaging increases treatment compliance, including medication adherence [8]; however, there is no previous research on the use of SMS text message-based digital communication on reducing rates of SSIs.

Many strategies, including preoperative antibiotic prophylaxis protocols, exist for preventing SSIs for elective surgery patients [9]. Decolonization is an antibiotic prophylaxis protocol in which patients apply intranasal mupirocin ointment and use chlorhexidine gluconate wash prior to surgery, resulting in decolonization of *Staphylococcus aureus.* Studies on the use of intranasal mupirocin ointment for the decolonization of *S. aureus* show reductions in SSIs [10-14]. Immerman et al [15] found that a protocol consisting of a 5-day course of nasal mupirocin and one preoperative chlorhexidine gluconate shower scrub resulted in decolonization in 61% to 72% of patients. Unfortunately, patient compliance for these procedures remains as low as 31.1% [16]. Patient compliance remains low for a number of reasons: (1) forgetting to use the products each day, (2) not understanding the instructions, (3) mistaking the frequency of application, or (4) not retrieving the prescription from the pharmacy. An automated reminder system can address many of these issues. Patients can be prompted to ensure that they have received their prescription and decolonization materials; they can also be sent daily reminder messages on when to use the decolonization materials.

To improve communication, some health care providers use electronic portals or apps, each of which has its own advantages and disadvantages. One disadvantage with apps and website-based systems is that the increased time for profile creation and app installation becomes a consistent usability concern [17]. Automated phone calls and text messages bypass such activities and remove complex barriers to implementation. In one meta-analysis, Kashgary et al [18] found that mobile interventions were able to increase medication adherence by 22%. This improvement in medication adherence suggests the potential for mobile interventions to significantly improve outcomes, streamline preoperative documentation, and lower long-term costs.

The purpose of this study was to investigate the feasibility of an automated intervention by focusing on patient response rates and satisfaction of using such a system. A decolonization protocol was previously implemented at Barnes-Jewish Hospital in St Louis, MO, for the orthopedic joint reconstruction service. Automated text messaging systems, named EpxDecolonization for preoperative messages and EpxWound for postoperative monitoring of pain and wound infections, were then implemented. The infrastructure for the implemented systems was provided by Epharmix, a startup company in St Louis, which named all its interventions with the prefix “Epx.”

We hypothesized that a telemedicine intervention in the form of automated text messages or phone call reminders would increase compliance with decolonization to prevent SSIs and effectively detect signs and symptoms of SSIs postoperatively to reduce unnecessary readmissions.

## Methods

### Procedure

This implementation was submitted to Washington University’s Institutional Review Board for review and was approved to be pursued as a quality improvement (QI) project. Patients undergoing primary joint reconstructions (hip and knee replacement) at an academic tertiary care facility (Barnes-Jewish Hospital) from November 29, 2015 to September 3, 2016 (data cutoff) were offered the option to enroll in the EpxDecolonization and EpxWound systems in addition to the standard perioperative care; some chose to be enrolled in only one system. Patients signed a consent form and provided a cellphone number or landline to be contacted at. To include as many patient populations as possible, such as older patients or those of lower socioeconomic status who may not be comfortable with texting or may not have access to smartphones, the systems were designed to enable usage with either text or voice calling capabilities. The only inclusion criterion was that the patient was undergoing an elective hip or knee replacement surgery. Patient responses were included in the analysis only if the entire session (EpxDecolonization or EpxWound) was completed by September 3, 2016.

Six days prior to their surgery, patients commenced with the EpxDecolonization system. EpxDecolonization sent texts or voice calls to ensure that patients received their decolonization supplies and, once procured, asked patients daily whether they had used their nasal ointment or chlorhexidine gluconate. When patients responded that they had not procured their decolonization materials, an alert was sent to the nurse in charge of their care. This information was recorded in the Epharmix system and could be checked by clinical staff, but the system did not generate an alert if a patient did not use their decolonization supplies to ensure that the number of alerts did not become a burden.

EpxWound sent texts or calls to patients to track pain and status of the wound. EpxWound was designed to identify SSIs between the patient’s surgery and their 2-week follow-up appointment. Thus, patients received daily messages from postoperative day 5 to 19 (15 consecutive days of messages) to cover a slightly longer time frame in case the patient’s 2-week follow-up appointment was delayed. Patients answered questions about their pain, wound status, and temperature. An alert was generated to the nurse in charge of their care in the event of increased redness, drainage, or odor, and if a fever was present.

**Figure 1:**
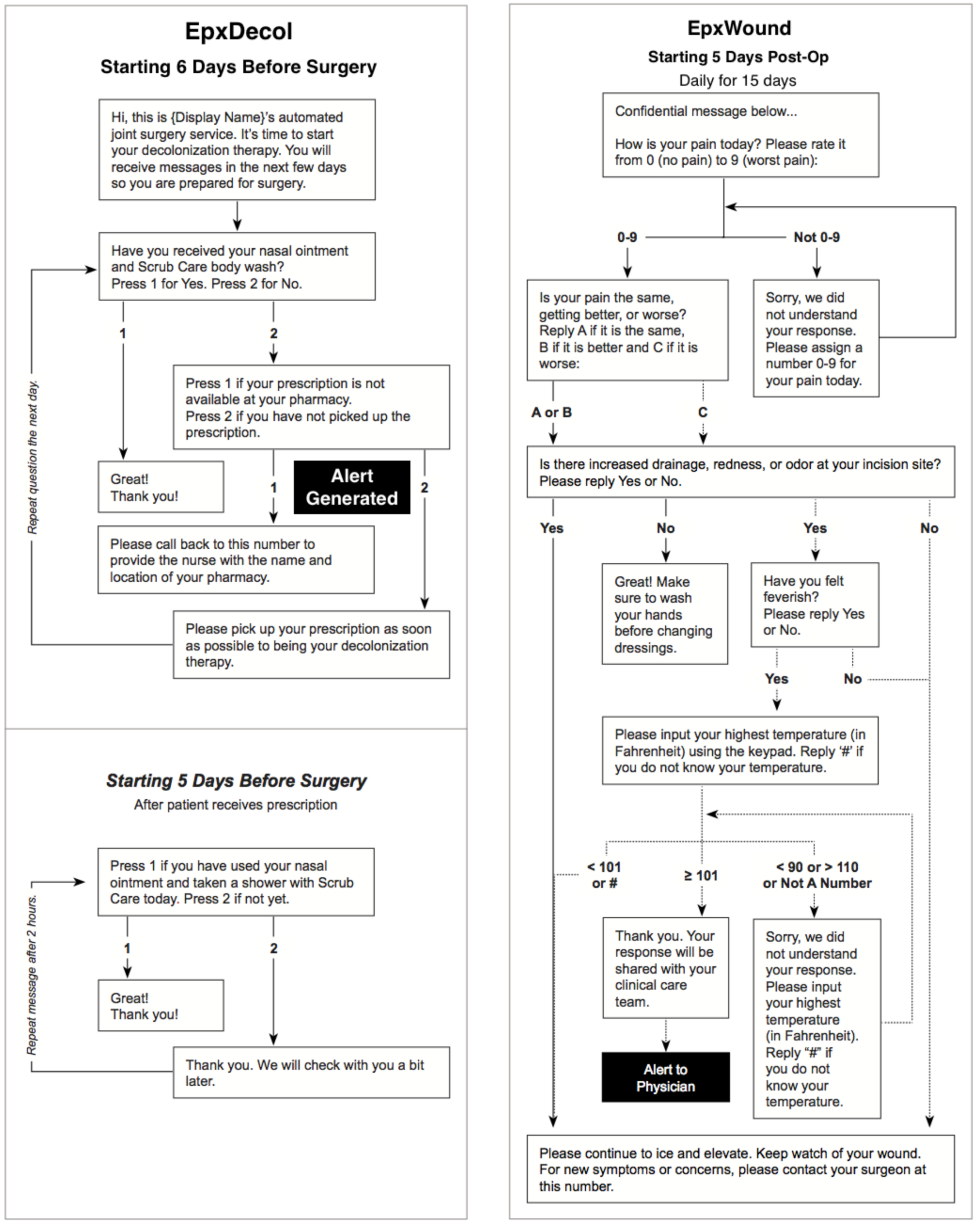
Text/Call algorithm for EpxDecolonization (EpxDecol) and EpxWound. In EpxDecolonization, patients were asked whether they had received/used their nasal ointment and body wash in two separate questions.

The preoperative EpxDecolonization system and postoperative EpxWound system are depicted in Figure 1. Alerts were sent to nurses either via automated email or phone calls. Following a generated alert, patients were contacted by a nurse within 2 hours or, if after hours, the following morning. Nurses who were responding to an alert called the patient to inquire about any further suggestions of an SSI or to ensure that the patient procured their decolonization supplies. Patients were asked to present to the clinic or were prescribed an antimicrobial if an SSI was suspected. Daily response rates for each patient were recorded throughout the study.

Following use of the systems, an automated electronic survey using a 1 to 9 response scale was delivered to assess the care delivered by the provider (On a scale of 1 to 9, how would you rate your care by your provider?), the number of messages they received (On a scale of 1 to 9, how do you feel about the number of messages you received through our service? [1=too few, 5=perfect amount, 9=too many]), and whether the EpxDecolonization and EpxWound improved communication with their doctors (On a scale of 1 to 9, do you think this service improved communication with your doctor? [1=significantly worsened, 5=no change, 9=significantly improved]). Only fully completed survey responses were included in our analysis (fewer patients responded to the survey than used the Epharmix systems).

The primary outcome was the daily response rate for all patients enrolled in a given week. Secondary outcomes were whether patients reported that EpxDecolonization and EpxWound improved communication, how many alerts were generated during the study, and how patients felt about the message frequency and overall care provided.

The algorithm and questions for the Epharmix systems were developed by medical students with the assistance of the joint reconstruction team. Software engineers at Epharmix (St Louis, MO, USA) coded the algorithm and created an enrollment platform on a Health Insurance Portability and Accountability Act (HIPPA)-compliant server. The system was then reviewed by the HIPPA compliance officer at Washington University.

### Participants

Participation was voluntary. Patient ages were not collected because we were not authorized to access patient health information. Enrollment was offered at a preoperative patient education joint replacement class. Attendance at the joint replacement class was required for all patients who had not received a joint replacement within the last 12 months.

### Statistical Analysis

Daily response rates for EpxDecolonization and EpxWound included all responses from patients who consented via the text message authorization sequence or via phone using the voice system. The proportion of sessions responded to each day of the intervention during the months of November 2015 to September 2016 was calculated by using the following formula: number of patients who responded to a text message or phone call on a particular day of the intervention over the 40-week study period divided by the total number of patients who received a text message or phone call on that same day of the intervention over the 40-week study period. The percentage of patients who responded at least once during that day was recorded.

Using the automated survey results, median, mean, and standard deviation scores for how the participants rated the overall quality of care and whether the system improved communication were calculated using Microsoft Excel. Median, mean, and standard deviation scores for frequency of messages were also calculated using Excel.

### Server

Epharmix maintains a mature stack on HIPAA-compliant servers at Washington University in St Louis. This stack allows maintenance personnel to focus on a single environment instead of having two separate environments, which can introduce more complexities. Updates and patches are more easily monitored and applied under this single environment.

Epharmix is hosted on servers provided by Armor, an industry-leading security-hosting provider that specializes in compliant hosting environments and offers advanced security services (eg, network perimeter defense, intrusion detection). For all the data Epharmix retains, the app stores them in secured, AES256-encrypted vaults that are managed by a role-based access control system. All connections to the Epharmix Web portal were encrypted via SSL/TLS so providers could access in a secure manner. Messages sent to patients were carefully designed; patient identifiers were removed from the content.

## Results

### Overview

At the end of the 40-week period, 638 and 642 patients were enrolled in EpxDecolonization and EpxWound, respectively. Approximately one-quarter of the patients chose the automated phone call intervention (27.6%, 176/638 for EpxDecolonization and 25.4%, 163/642 for EpxWound). The remaining three-quarters chose text messages (72.4%, 462/638 for EpxDecolonization and 74.6%, 479/642 for EpxWound). The proportion of total sessions responded to was 85.2% for EpxDecolonization and 78.4% for EpxWound. The surgical site infection rate for hip and knee replacement during our study period was 0.8%.

### Daily Response Rates and Enrollment

**Figure 2:**
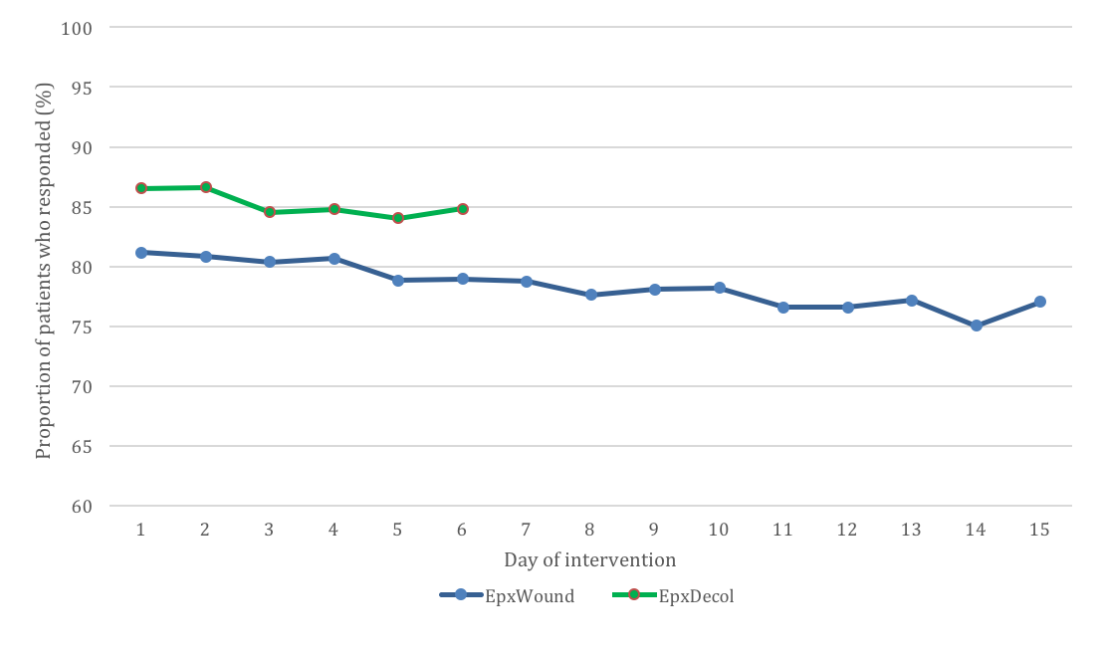
The proportion of sessions responded to during each day of the intervention over the 40-week trial period for the EpxDecolonization (6 intervention days) and EpxWound (15 intervention days) programs.

For EpxDecolonization, the proportion of sessions responded to decreased from 86.5% (552/638) on the first day to 84.0% (526/626) on the second-to-last day (Figure 2). For EpxWound, the proportion of sessions responded to decreased from 81.2% (521/642) on the first day to 75.0% (466/621) on the second-to-last day (Figure 2). Due to limitations with the QI project implementation, we could not obtain the number of patients who declined enrollment in the study. However, nurses responsible for enrollment in the study estimated to us that more than 95% of patients enrolled. These nurses also indicated that the primary reason for not enrolling was that the patient did not believe that the system was necessary for their care.

**Figure 3:**
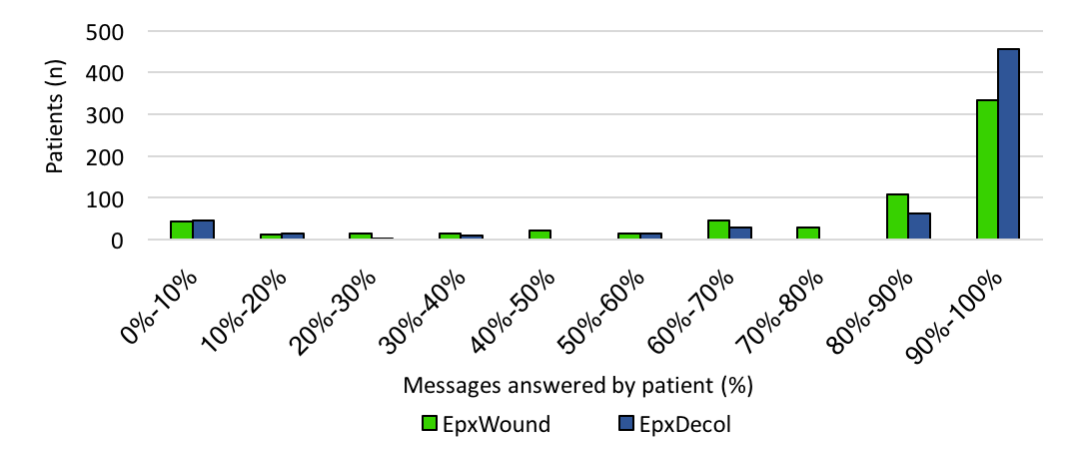
The distribution of percentage of sessions answered by patients enrolled in the EpxDecolonization (EpxDecol) and EpxWound interventions.

As shown in Figure 3, 71.3% (455/638) of EpxDecolonization patients and 52.0% (334/642) of EpxWound patients responded to 90% to 100% of messages.

### Dropout Rate

The dropout rate, defined as the percentage of patients who requested to stop receiving text messages, was 2.0% (13/638) for EpxDecolonization and 3.7% (24/642) for EpxWound (Multimedia Appendix 1). The greatest number of dropouts occurred on day 4 for EpxDecolonization (6 patients dropped out) and on day 1 for EpxWound (6 patients dropped out).

### Alerts

**Figure 4:**
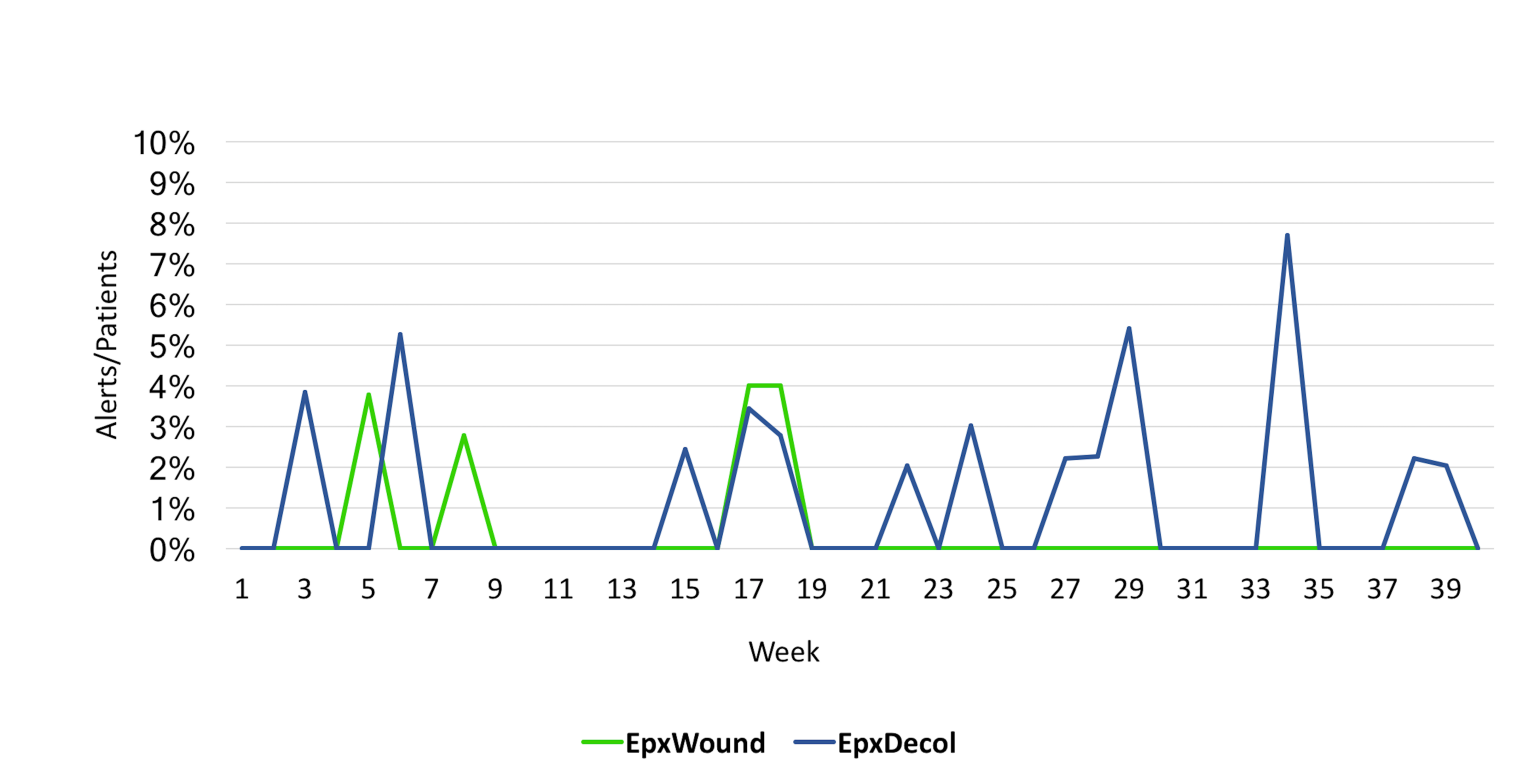
Percentage of patients that triggered an alert each week using EpxDecolonization (EpxDecol) and EpxWound over the 40-week trial period.

Figure 4 shows that the percentage of patients who triggered an alert in a given week never exceeded 8% for either system; the proportion of patients that generated an alert over the 40-week period was 1.1% (7/642) for EpxWound and 1.9% (12/638) for EpxDecolonization. Twelve alerts were generated for EpxDecolonization and seven for EpxWound. All 12 alerts from EpxDecolonization were triggered because the patient had not procured their decolonization supplies. The EpxDecolonization system was not designed to alert the medical team if the patient had not completed their decolonization procedure. The patient decolonization completion record was available for viewing in the Epharmix portal. For ExpWound, three alerts were generated for increased redness, odor, and drainage, and four for increased redness, odor, and drainage with fever. Nurses called each of these patients within 2 hours of the generated alert or, if after hours, the next business day. Once contacted by a nurse, the intervention continued for each patient that generated an alert.

### Survey Results

**Figure 5:**
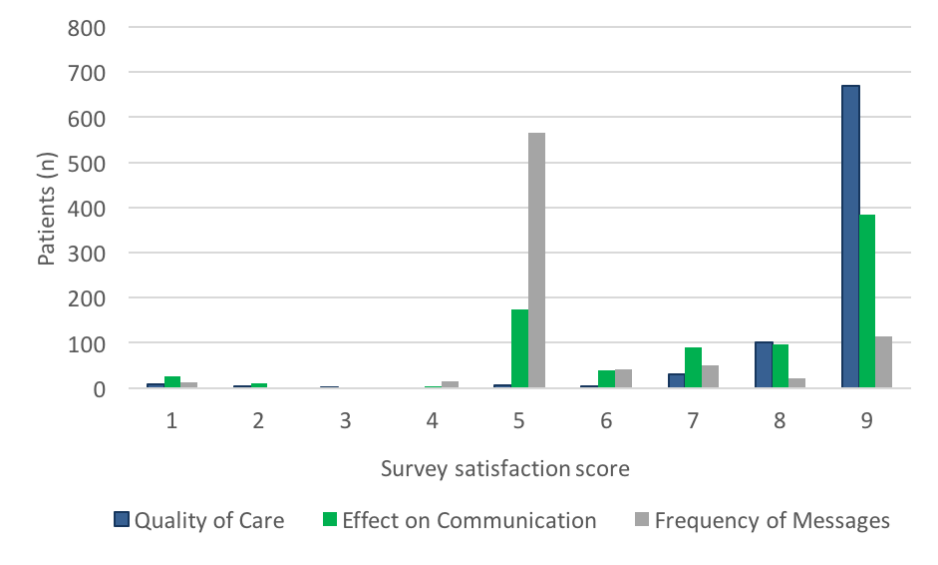
Patient satisfaction with EpxDecolonization and EpxWound. Patients rated their care provided by their medical care team on a scale from 1 to 9 (1=terrible, 5=average, 9=excellent), whether EpxDecolonization and EpxWound improved communication with their doctor (1=significantly worsened, 5=no change, 9=significantly improved), and their satisfaction with the number of messages that they received (1=too few, 5=perfect amount, 9=too many).

For the combined 1280 EpxWound and EpxDecolonization sessions, 821 (64.14%) postoperative satisfaction surveys were fully completed. One survey was sent for each session and because patients could be enrolled in one or both systems, patients were able to complete one or two surveys. When asked about the overall care provided during this study, patients reported a median score of 9 out of 9 (mean 8.6, SD 1.1), as shown in Figure 5. The overwhelming majority (97.0%, 796/821) of patients rated the overall quality of their care as 6 out of 9 or higher. Patients reported a median score of 8 out of 9 (mean 7.3, SD 2.1) when asked if Epharmix improved communication with the care team (Figure 5). The majority of patients (69.9%, 566/821) reported that the system improved their communication.

The median satisfaction score for the number of messages sent was 5 (best possible) and mean 5.7 (SD 1.6) (Figure 5). The majority of patients (68.9%, 566/821) felt that the systems sent out the perfect number of messages (rating of 5). However, a subset (26.9%, 221/821) of patients reported that too many messages were sent (rating of >5), and a smaller (4.2%, 34/821) subset indicated that not enough messages were sent (rating of <5).

## Discussion

### Principal Findings

Overall, we report high total response rates (85.2% and 78.4% for EpxDecolonization and EpxWound, respectively); high satisfaction scores (median values of 9, 8, and 5 [perfect score] for patient-rated care, improvement in communication, and number of messages received, respectively); and a low dropout rate (2.0%, 13/638 for EpxDecolonization and 3.7%, 24/642 for EpxWound) for both automated phone and SMS text messaging systems.

Historically, the perioperative surgical management of patients comprised of unsupported patients self-monitoring their own care status (based on discussions with providers). Patients were expected to recall and implement the prescribed perioperative protocol correctly and providers had to hope for compliance. On discharge, health care providers relied on patients for symptom monitoring and alerting their providers in a timely manner when issues arose in addition to the scheduled postoperative clinic visit. Our system has the potential to facilitate better patient self-monitoring and provides a new way for patients to communicate the results to their health care providers. These communications could include first signs of infection as well as a notification that the patient has not yet received decolonization materials.

Our study demonstrates that EpxDecolonization and EpxWound are effective at reaching patients and facilitating patient self-monitoring of SSI prevention and identification, as concluded from high response rates. Also, user survey data shows high satisfaction with each system. Specifically, patients reported that the Epharmix systems sent the appropriate number of messages and that the systems improved communication with their provider. These positive impressions likely contributed to the high response rates. Our promising findings with these systems suggest potential for use in broader applications.

Text message interventions offer advantages over more traditional interventions, such as nurses calling patients. Text messages can be sent in the morning and the patients can respond at their own convenience. When a nurse calls, the patient must be available to speak at that moment. The difficulty that nurses have getting in contact with patients via a phone call is a documented dilemma. Bebko et al [9] reported that despite three attempts, nurses could not reach over 28% (31/110) of patients after hospital discharge. Our interventions primarily used text messages; therefore, this increased time frame for patient response may have contributed to our high response rates.

Another potential domain of enhanced telemedicine approaches is improving patient satisfaction. Patient satisfaction is becoming increasingly important. Systems such as EpxDecolonization and EpxWound may play a critical role in improving patients’ rating of overall care. This is partly captured by the 9 out of 9 median rating for the overall care provided. A potential component of that highly rated provided care could be explained by the patients’ 8 out of 9 median rating that the Epharmix systems improved communication with the health care team.

In our results, we found that EpxDecolonization had a higher response rate than EpxWound. This difference could be explained by the fact that EpxDecolonization was preoperative whereas EpxWound was postoperative and that EpxDecolonization had fewer questions than EpxWound. EpxWound and EpxDecolonization suggested that automated communication systems could elicit high patient response rates during the critical perioperative period. The high response rates also demonstrate ease of use because there are other forms of communication that could serve the same purpose of communication, but presumably put more burden on the respondent [18].

Although response rates were high, patient engagement decreased over the length of the study. A small percentage of patients dropped out (2.0%, 13/638 for EpxDecolonization and 3.7%, 24/642 for EpxWound) and response rates decreased (1.7% decrease for EpxDecolonization and 4.1% decrease for EpxWound). This usage fatigue is well demonstrated in other studies [19,20].

Despite the largely positive responses, approximately 20% of patients (Figure 5) did not feel the system affected their communication, and a very small subset indicated that the systems worsened their communication. After talking to the nurses, a potential explanation may be that these patients generated an alert but did not receive prompt follow-up by the nurse receiving the alert. This emphasizes the importance of medical staff implementing robust processes to ensure that patients obtain prompt follow-up after triggering the system. Due to the limitations of this study, no further investigation into the patient demographics or patient situations could be pursued. Another reason could be the patients were already diligent about medication compliance and wound monitoring, and felt that our system added little to no value to their experience. Future studies will aim to better understand the reasons why certain patients felt that the system made no difference or even worsened the communication with the health care team.

Patient survey data showed that patients were inclined to use our system. At the same time, because the system is automated, it improved communication (based on patient-rated results) without putting a significant burden on surgical group employees. Providers reported that the system was convenient because it required minimal work for them to enroll and was efficient at monitoring patients. Further, they were assured that their patients were being tracked perioperatively and knew that they would be alerted to patients who needed extra attention. In terms of cost measures and savings, surgical groups who pay staff to check in on patients by phone may be able to save in this area, especially because many surgeries are becoming reimbursed by bundled payments that will not reimburse for individual aspects of care delivery. A study conducted by Semple et al [21] that implemented an app to monitor surgical sites post-breast reconstruction or orthopedic surgery found similar high satisfaction rates among patients and providers.

Follow-up conversations with the nursing staff and surgeons indicated that the number of alerts was within manageable limits for the health care team. It is also notable that 1.1% of patients triggered an EpxWound alert, which is reflective of the observed SSI rate of the clinic (0.8%) in which the QI project was conducted. This adds additional validity to our observations. Even with this low alert rate, three of the seven total alerts for EpxWound were triggered by the same patient, highlighting the ability of Epharmix interventions to track in-need patients until all their complications are addressed. These data indicate that EpxDecolonization and EpxWound patient alerts are manageable for the nursing staff without creating an excessive work burden.

We also tried to determine whether either of our systems was sending automated messages too frequently or not frequently enough. Approximately 70% of patients reported that the number of messages was just right. Due to the scaling of previous questions, a number of patients commented at the end of the survey that they had mistakenly selected 9 for this question instead of 5. This issue may explain some of the patients who reported that there were sent too many messages, and this can be easily modified when designing future survey questions.

As bundled payments become more prevalent, providers will bear most of the cost of postoperative complications. Given the numerous Enhanced Recovery After Surgery initiatives across the United Stats aimed at decreasing postoperative complications while maximizing use of resources, automated communication systems such as EpxDecolonization and EpxWound are uniquely poised to facilitate these cost-reducing measures in a standardized and patient-centered way. Effort is currently being focused on integrating the EpxWound and EpxDecolonization systems into existing electronic medical record platforms. Additionally, the technology used to build EpxDecolonization and EpxWound is currently being expanded to other surgical specialties including but not limited to cardiothoracic, colorectal, neurosurgery, trauma, and urology to have a broader impact on improving overall surgical care.

### Limitations

Limitations of the study related to the QI status of the project, the voluntary enrollment structure, and the lack of a concurrent control group. Because this was an early QI study to assess feasibility, we were unable to measure the clinical effectiveness of these automated systems that we hope to study in the future. EpxDecolonization encouraged patients to procure their prescriptions and solicited daily responses on whether their ointment and chlorhexidine gluconate was used. However, we were unable to investigate significant improvements in decolonization compliance because this QI project did not include a mechanism to objectively assess decolonization compliance beyond the patient-reported responses. We were also unable to determine the percentage of patients who procured their decolonization supplies after a generated alert from the EpxDecolonization system and nurse intervention. Also, due to the QI status of this project, we were not permitted to obtain and evaluate the number of patients that declined enrollment in our study and the number of patients who underwent a knee replacement versus a hip replacement surgery. We were also limited by the amount of follow-up and patient interviewing that we were able to conduct. For example, it would have been instructive to investigate the reasons for the small subset of patients who responded to 0% to 10% of messages, but it was not within the scope of the QI project. The voluntary enrollment structure of this study provides another limitation. It is possible that those who were willing to consent were more likely to respond to inquiries from the automated systems. Another limitation was that there was no concurrent control group without the Epharmix interventions. Future studies will incorporate this type of follow-up to provide maximal opportunity for improvement. The studies will also investigate the specificity and sensitivity of the systems’ alerts, because any new tool for treatment should be assessed for reliability and validity [22]. With further data on specificity and sensitivity, we can assess the efficacy of EpxDecolonization improving decolonization compliance and EpxWound in detecting SSIs earlier.

### Conclusions

In summary, we developed automated SMS text messaging and calling systems called EpxDecolonization and EpxWound in an effort to improve perioperative care in patients undergoing orthopedic joint reconstruction. Our project demonstrated that patients responded to 85.2% and 78.4% of all sessions sent by EpxDecolonization and EpxWound, respectively. The majority of patients felt that the Epharmix systems improved communication with their providers and sent out the appropriate number of messages. From discussions with providers, surgeons and nurses readily adopted the systems, and most patients were interested in using the system. The automated text or phone call systems, EpxDecolonization and EpxWound, were shown to be proactive tools that are not overly burdensome and have the potential to improve perioperative care within orthopedics and other surgical fields in a cost-effective manner. Although the QI status of this project limited our ability to correlate responses with patient outcomes, this will be addressed in future studies. These studies will also assess quality metrics as well as the sensitivity and specificity of the generated alerts by EpxDecolonization and EpxWound.

## Appendix

Multimedia Appendix 1 Number of patients receiving text messages/phone calls during each day of the EpxDecol and EpxWound interventions.

## Abbreviations

QI: quality improvement
SMS: short message service
SSI: surgical site infection
