# Supplementary figures and images for: Accessible Communication Tools for Surgical Site Infection Monitoring and Prevention in Joint Reconstruction: Feasibility Study
Source: JMIR Perioper Med. 2018 Jan 17;1(1):e1. doi: 10.2196/periop.7874 (PMC7709860; doi:10.2196/periop.7874)

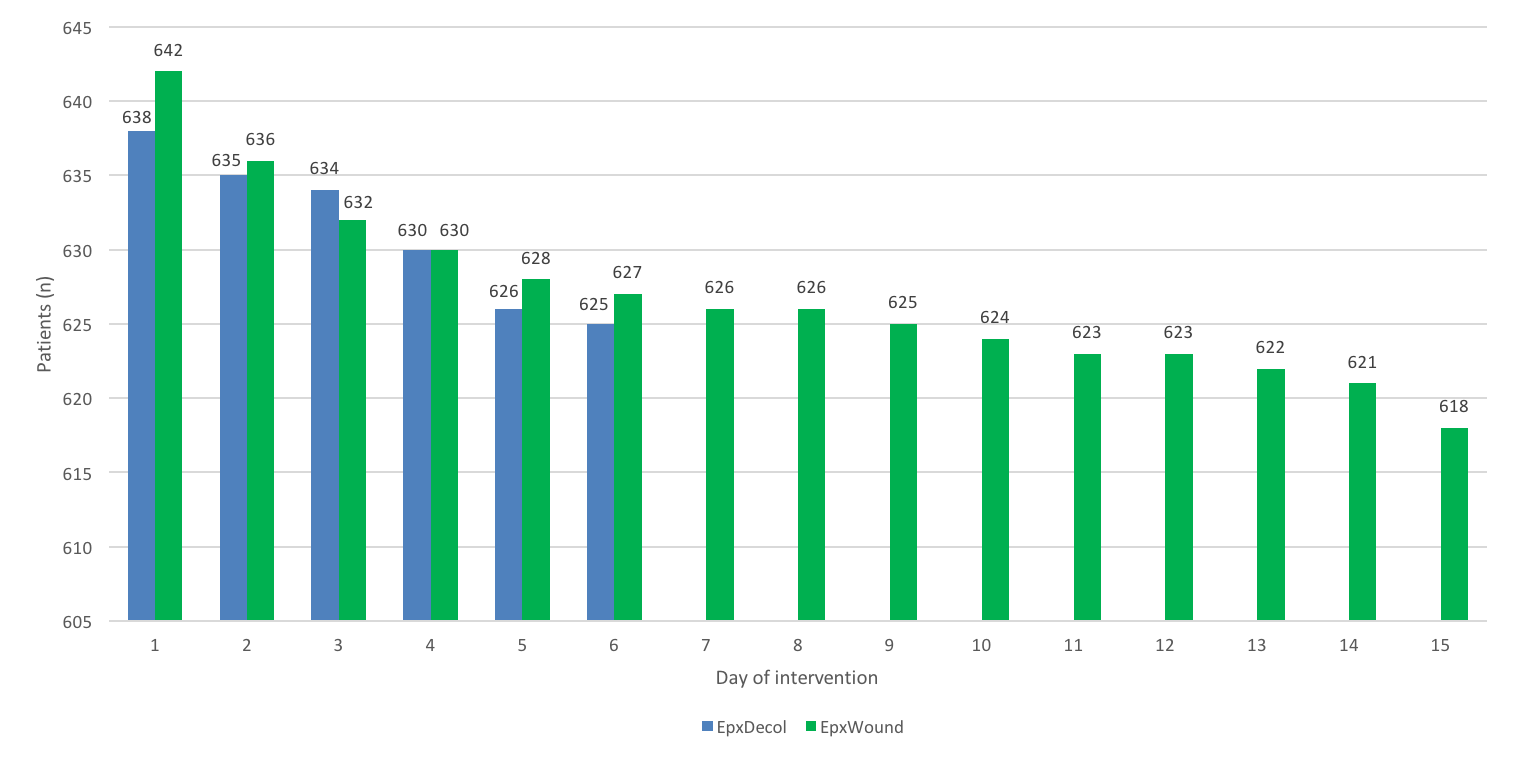

Supplement: Multimedia Appendix 1 [file periop_v1i1e1_app1.png]
